# 数字PCR与实时荧光定量PCR检测慢性髓性白血病患者BCR::ABL mRNA水平的比较性研究

**DOI:** 10.3760/cma.j.issn.0253-2727.2023.11.004

**Published:** 2023-11

**Authors:** 汉林 高, 玥 郝, 文敏 陈, 玲娣 李, 旭 王, 亚溱 秦, 倩 江

**Affiliations:** 北京大学人民医院、北京大学血液病研究所，北京 100044 Peking University People's Hospital, Peking University Institute of Hematology, Beijing 100044, China

**Keywords:** 白血病，髓样，慢性, 实时定量聚合酶链反应, 数字聚合酶链反应, 融合蛋白质类，BCR::ABL, Leukemia, myeloid, chronic, Real-time quantitative polymerase chain reaction, Digital polymerase chain reaction, Fusion proteins, BCR::ABL

## Abstract

**目的:**

比较数字PCR（dPCR）与实时荧光定量PCR（qPCR）方法检测慢性髓性白血病（CML）患者外周血BCR::ABL（P210）mRNA水平的差异性、相关性与一致性。

**方法:**

本研究为非干预性、横断面研究。收集2021年9月至2023年2月在北京大学人民医院就诊的服用酪氨酸激酶抑制剂（TKI）至少获得完全细胞遗传学反应（CCyR）的CML患者同时采用dPCR和qPCR方法检测 BCR::ABL mRNA水平的结果，分别采用Wilcoxon符号秩检验、Spearman相关系数、Bland-Altman分析比较两种方法的差异性、相关性、一致性。

**结果:**

356例CML患者的459对外周血样本分别采用dPCR与qPCR方法检测BCR::ABL mRNA水平的结果用于分析。总体上，两种方法检测结果差异有统计学意义（*P*<0.001）。根据分子学反应分层分析，这种差异仅存在于获得≥分子学反应4.5（MR4.5）（*P*<0.001）后，而非未达主要分子学反应（MMR）（*P*＝0.922）、MMR（*P*＝0.723）和分子学反应4（MR4）（*P*＝0.099）水平。总体上，dPCR与qPCR方法检测BCR::ABL mRNA水平具有中度相关性（*r*＝0.761，*P*<0.001）。但随着分子学反应加深，这种相关性逐渐减弱甚至消失：未达MMR（*r*＝0.929，*P*<0.001），MMR（*r*＝0.815，*P*<0.001），MR4（*r*＝0.408，*P*<0.001），MR4.5（*r*＝0.176，*P*＝0.176）。一致性上，MR4.5组两种方法检测结果的一致性弱于其他各组：未达MMR（*d*＝0.042，*P*＝0.846），MMR（*d*＝0.054，*P*＝0.229），MR4（*d*＝−0.020，*P*＝0.399），MR4.5（*d*＝−0.219，*P*<0.001）。

**结论:**

CML患者当获得深层分子学反应后，更适合采用dPCR方法监测BCR::ABL（P210）mRNA水平以提高精准性。

## Abstract (en)

**Objective:**

To compare digital polymerase chain reaction（dPCR）and real-time quantitative PCR（qPCR）measurements of BCR::ABL（P210）mRNA expression in patients with chronic myeloid leukemia（CML）.

**Methods:**

In this non-interventional, cross-sectional study, BCR::ABL（P210）mRNA was simultaneously measured by dPCR and qPCR in peripheral blood samples collected from patients with CML who underwent tyrosine kinase inhibitor therapy and who achieved at least a complete cytogenetic response from September 2021 to February 2023 at Peking University People's Hospital. The difference, correlation, and agreement between the two methods were evaluated using the Wilcoxon signed-rank test, Spearman's correlation, and Bland-Altman analysis, respectively.

**Results:**

In total, 459 data pairs for BCR::ABL mRNA expression measured by dPCR and qPCR from 356 patients with CML were analyzed. There was a significant difference in BCR::ABL mRNA expression between the two methods（*P*<0.001）. When analyzed by the depth of the molecular response（MR）, a significant difference only existed for patients with ≥MR4.5（*P*<0.001）. No significant difference was observed for those who did not achieve a major MR（no MMR; *P*＝0.922）or for those who achieved a major MR（MMR; *P*＝0.723）or MR4（*P*＝0.099）. There was a moderate correlation between the BCR::ABL mRNA expression between the two methods（*r*＝0.761, *P*<0.001）. However, the correlation gradually weakened or disappeared as the depth of the MR increased（no MMR: *r*＝0.929, *P*<0.001; MMR: *r*＝0.815, *P*<0.001; MR4: *r*＝0.408, *P*<0.001; MR4.5: *r*＝0.176, *P*＝0.176）. In addition, the agreement in BCR::ABL mRNA expression between the two methods in those with MR4.5 was weaker than other groups（no MMR: *d*= 0.042, *P*＝0.846; MMR: *d*＝0.054, *P*＝0.229; MR4: *d*＝−0.020, *P*＝0.399; MR4.5: *d*＝−0.219, *P*<0.001）.

**Conclusion:**

dPCR is more accurate than qPCR for measuring BCR::ABL（P210）mRNA expression in patients with CML who achieve a stable deep MR.

实时荧光定量聚合酶链反应（qPCR）检测BCR::ABL mRNA水平已成为慢性髓性白血病（CML）的标准方法，可用于评估分子学反应、监测微小残留病变（MRD）水平[1]，并指导后续治疗。目前，停药是CML患者的重要追求目标，达到稳定的深层分子学反应（DMR）是停药的门槛，精准的BCR::ABL mRNA水平评估是停药的必要条件。数字PCR（dPCR）是近年新兴的能够提供BCR::ABL融合基因序列绝对数量的技术，国外已将dPCR技术应用于CML患者MRD检测，提高了敏感性和准确性[2]–[4]。dPCR通过将样本分散到几千甚至上万的液滴中进行单终点PCR，不受到抑制剂、引物效率或校准样品的影响，也不需要标准参考曲线，能够给出绝对定量的结果[5]。这些特点使其能够克服qPCR在检测BCR::ABL mRNA时只能给出相对定量的结果的局限性。如今，dPCR技术的可及性已经大大提升，目前dPCR技术是应用于达到DMR的患者中还是作为监测CML患者分子学反应的常规工具，国外学者的意见尚不统一。虽然dPCR技术监测MRD的准确性高于qPCR技术，但操作技术更为复杂，检测成本和检测费用明显增高。因此，何时应用以及哪些人更适合应用dPCR技术监测MRD水平值得探讨。本研究比较了356例CML患者的459对外周血标本采用qPCR与dPCR检测BCR::ABL mRNA水平的差异性、相关性以及一致性，以探讨选择使用dPCR方法监测CML患者MRD的最佳时机。

## 病例与方法

一、研究设计

本研究为非干预性、横断面研究，收集2021年9月至2023年2月在我院就诊的服用酪氨酸激酶抑制剂（TKI）至少获得完全细胞遗传学反应（CCyR）的CML患者采用dPCR以及qPCR检测 BCR::ABL融合基因的结果。

二、方法

对患者的外周血标本提取RNA逆转录为cDNA后，通过常规qPCR方法[6]检测患者BCR::ABL（P210）mRNA水平，再转换成BCR::ABL/ABL（％）国际标准化（IS）值。采用白血病融合基因BCR::ABL（P210）核酸检测试剂盒（数字PCR法）（思纳福公司产品）实施dPCR检测。该试剂盒采用RNA样本，同时检测e13a2和e14a2两种转录本类型，BCR::ABL融合基因（P210）和ABL基因特异性引物和探针分别用FAM和VIC荧光标记，在DQ24-Dx（思纳福公司产品）数字PCR一体机上一管内进行扩增，反应条件为：50 °C逆转录30 min，95 °C预变性5 min，然后先进行5个96 °C15 s，60 °C20 s的循环，再进行40个96 °C 15 s，63 °C 20 s的循环，最后72 °C保持2 min。通过泊松分布计算样本中BCR::ABL和ABL的RNA拷贝数，计算BCR::ABL和ABL拷贝数的比值并换算成IS值。分子学反应参考《慢性髓性白血病中国诊断与治疗指南（2020年版）》中的定义[7]。

三、统计学处理

描述性分析结果以中位数（范围）或数字（百分比）表示。使用Wilcoxon符号秩检验比较dPCR和qPCR方法检测的BCR::ABL/ABL（％）IS值的差异性，全部选择双侧检验。使用Spearman相关系数评估dPCR和qPCR方法检测的BCR::ABL/ABL（％）IS值进行以10为底对数变换所得结果之间的相关性（dPCR与qPCR方法均可检出BCR::ABL融合基因），对Spearman相关系数的检验，全部选择单侧检验。使用Bland-Altman分析方法评估dPCR和qPCR方法检测的BCR::ABL/ABL（％）IS值进行以10为底对数变换所得结果的一致性（dPCR与qPCR方法均可检出BCR::ABL融合基因）。一致性分析差值*d*的计算公式为*d*＝log（dPCR）-log（qPCR）。两种方法检测的BCR::ABL mRNA水平的差值均值*d*与95％一致性界限（Limits of agreement，LoA）能够反映两种方法检测结果的一致程度。差值均值*d*的绝对值越大，说明两种方法测量结果相差越大，一致性越弱，*P*<0.05为差异有统计学意义。上述统计分析均根据qPCR分子学反应分层后进行。所有统计分析均采用R 4.2.2软件进行。

## 结果

一、病例特征

本研究纳入356例CML患者，患者临床特征见表1。318例患者分别进行了1次（288例）、2次（51例）、3次（16例）、4次（1例）、5次（1例）和6次（2例）检测，共获得459对外周血样本qPCR及dPCR检出的BCR::ABL mRNA水平结果。根据qPCR结果，包括分子学反应未达主要分子学反应（MMR）9例10对、MMR 20例21对、分子学反应4（MR4）46例57对、分子学反应4.5（MR4.5）25例31对和基因转阴256例340对。

**表1:** 356例慢性髓性白血病患者临床特征

特征	数值
性别[例（%）]	
男	216（60.7）
女	140（39.3）
年龄[岁，*M*（范围）]	51（11~88）
慢性期[例（%）]	354（99.4）
总TKI服用时间[年，*M*（范围）]	6.5（0.1~23.6）
目前用药[例（%）]	
一代TKI	214（60.1）
二代TKI	105（29.5）
三代TKI	22（6.2）
停药	15（4.2）
目前治疗反应[例（%）]	
未达MMR	9（2.5）
MMR	20（5.6）
MR4	46（12.9）
MR4.5	25（7.0）
基因转阴	256（71.9）

注 TKI：酪氨酸激酶抑制剂；MMR：主要分子学反应；MR4：分子学反应4；MR4.5：分子学反应4.5

全部患者的中位BCR::ABL/ABL（％）IS值通过dPCR和qPCR方法检测结果分别为0.0011％（0～1.4000％）和0（0～0.9500％）（*P*<0.001）。117对样本（25.5％）2种方法均检出BCR::ABL mRNA，174对（37.9％）均未检出BCR::ABL mRNA，2对（0.4％）dPCR方法检测不到而qPCR方法可以检出，166对（36.2％）样本相反。

二、差异性

基于qPCR检测结果的分子学反应，我们将356对样本的BCR::ABL/ABL（％）IS值进行分组以比较差异性。总体上，356对样本的两种方法检测的BCR::ABL mRNA水平差异有统计学意义（*P*<0.001）。根据qPCR检测结果评估分子学反应，并分层分析dPCR与qPCR检测的结果：未达MMR组（*P*＝0.922）、MMR组（*P*＝0.723）和MR4组（*P*＝0.099）的BCR::ABL mRNA水平差异均无统计学意义，MR4.5组（*P*<0.001）和基因转阴组（*P*<0.001）则差异均有统计学意义（表2）。

**表2:** 不同分子学反应下数字PCR（dPCR）与实时定量PCR（qPCR）方法检测的BCR::ABL mRNA的差异性和相关性

分子学反应（基于qPCR法检测）	差异性				相关性		
	样本数（对）	dPCR法［％，*M*（范围）］	qPCR法［％，*M*（范围）］	*P*值	样本数（对）	*r*值	*P*值
未达MMR	10	0.2650（0.0370~1.4000）	0.3300（0.1100~0.9500）	0.922	10	0.929	<0.001
MMR	21	0.0280（0.0081~0.1500）	0.0320（0.0110~0.0970）	0.723	21	0.815	<0.001
MR4	57	0.0072（0~0.0360）	0.0060（0.0034~0.0098）	0.099	56	0.408	<0.001
MR4.5	31	0.0037（0~0.0150）	0.0023（0.0010~0.0032）	<0.001	30	0.176	0.176
基因转阴	340	0（0~0.0097）	0	<0.001			

总体	459	0.0011（0~1.4000）	0（0~0.9500）	<0.001	117	0.761	<0.001

注 MMR：主要分子学反应；MR4：分子学反应4；MR4.5：分子学反应4.5

三、相关性

基于qPCR检测结果的分子学反应，我们将117对两种方法均可检出BCR::ABL mRNA的数据进行分组，以比较相关性。总体上，117对样本的两种方法检测的BCR::ABL mRNA水平存在中度相关性（*r*＝0.761，*P*<0.001）。根据qPCR检测结果评估分子学反应，并分层分析两种方法检测的BCR::ABL mRNA水平：未达MMR组（*r*＝0.929，*P*<0.001）和MMR组（*r*＝0.815，*P*<0.001）两种方法检测结果存在高度相关性，MR4组仅有低度相关性（*r*＝0.408，*P*<0.001），MR4.5组无显著相关性（*r*＝0.176，*P*＝0.176）（表2）。

四、一致性

我们将117对两种方法均可检出BCR::ABL mRNA水平的结果，应用Bland-Altman方法分析，基于qPCR检测结果的分子学反应进行分组，比较dPCR与qPCR结果的一致性。总体上，117对样本的两种方法检测的BCR::ABL mRNA水平的差值均值*d*＝−0.052，*P*＝0.043，95％ LoA为（−0.655，0.550）。根据qPCR检测结果作基于分子学反应的分层分析：未达MMR组中10对样本的差值均值*d*＝0.042，*P*＝0.846，95％LoA为（−0.446，0.530）。MMR组中21对样本的差值均值*d*＝0.054，*P*＝0.229，95％LoA为（−0.355，0.463）。MR4组中56对样本的差值均值*d*＝−0.020，*P*＝0.399，95％LoA为（−0.648，0.609）。MR4.5组中30对样本的差值均值*d*＝−0.219，*P*<0.001，95％LoA为（−0.811，0.373）（表3）。MR4.5组的差值均值*d*的绝对值大于其他各组，且*P*<0.001，表明该组一致性较其他组弱。总体和各组的Bland-Altman图见图1。

**表3:** 不同分子学反应下数字PCR（dPCR）与实时定量PCR（qPCR）方法检测的BCR::ABL mRNA的一致性

分子学反应（基于qPCR法检测）	一致性				
	样本数（对）	*d*（95%*CI*）	*P*值	95%LoA下限（95%*CI*）	95%LoA上限（95%*CI*）
未达MMR	10	0.042（−0.112, 0.196）	0.846	−0.446（−0.713, −0.179）	0.530（0.263, 0.797）
MMR	21	0.054（−0.035, 0.143）	0.229	−0.355（−0.510, −0.200）	0.463（0.309, 0.618）
MR4	56	−0.020（−0.104, 0.064）	0.399	−0.648（−0.794, −0.503）	0.609（0.463, 0.754）
MR4.5	30	−0.219（−0.327, −0.111）	<0.001	−0.811（−0.999, −0.624）	0.373（0.186, 0.560）

总体	117	−0.052（−0.108, 0.003）	0.043	−0.655（−0.751, −0.558）	0.550（0.453, 0.646）

注 MMR：主要分子学反应；MR4：分子学反应4；MR4.5：分子学反应4.5

**图1:**
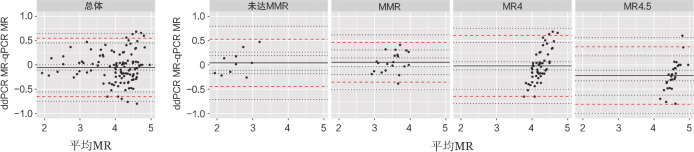
数字PCR（dPCR）与实时定量PCR（qPCR）方法一致性分析Bland-Altman图 注 MMR：主要分子学反应；MR4：分子学反应4；MR4.5：分子学反应4.5；黑色实线表示两种方法检测的BCR::ABL mRNA水平的差值均值*d*，上下两条红色虚线分别表示95％ LoA上下限，这三条线各自周围上下两条黑色点线之间的部分分别为差值均值*d*和95％ LoA上下限的95％置信区间

## 讨论

我们比较了dPCR与qPCR检测BCR::ABL（P210）mRNA水平的差异性、相关性和一致性发现：差异性上，两种方法的结果存在差异性，主要体现在获得≥MR4.5的患者中；相关性上，两种方法检测结果在分子学反应深度达到MR4时很弱，≥MR4.5时无相关性；一致性上，MR4.5组的差值均值*d*的绝对值明显大于其他各组（*P*<0.001），表明该组中两种方法检测结果的一致性弱于其他各组。这些结果提示，dPCR方法更适合在达到DMR的患者中应用，因为在这部分人群中，dPCR方法与传统的qPCR方法得出的结果的差异较大。

TKI停药已成为CML患者追求的重要目标，而停药的必要条件是获得稳定持久的DMR，分子学反应越深、越长，丧失MMR的机会越低，停药的成功率越高。dPCR方法检测MRD的准确性和敏感性优于传统qPCR方法，使其更适合应用于停药前和停药期间的监测。由于dPCR检测操作复杂，成本较高，最终导致检验费用较高。因此，过早地使用dPCR方法不仅无法提高检测的准确度，反而增加了患者的经济负担。相反，达到qPCR评估的DMR后再使用dPCR方法定期进行监测，可以更精准地评估BCR::ABL mRNA水平，从而发挥dPCR方法最大的价值。多项研究表明，dPCR方法有助于CML患者的BCR::ABL分子学反应监测以及未来实现和维持CML患者无治疗缓解的长期监测[8]–[13]。因此，dPCR方法更适合应用于达到DMR的患者，为其安全地停药做充分的准备。

本研究存在以下不足之处：样本量有限，特别是在未达MMR和MMR组中，由于这两组患者qPCR分子学反应较浅，在临床上不常进行dPCR检测，故样本量较少。

综上所述，本研究发现整体上dPCR与qPCR两种方法检测CML患者外周血BCR::ABL（P210）mRNA水平结果有差异，特别是在MR4.5组和基因转阴组中。提示临床医师在对CML患者分子学反应监测方法做选择时，建议在qPCR分子学反应达到稳定的DMR后再选用dPCR方法，也建议在进行TKI减量或停药的患者中应用dPCR方法，以提高分子学反应监测的精准度。
